# Dr. Hamilton’s Surgical Dispensary at the Medical College, 1848

**Published:** 1848-04

**Authors:** 


ART. II.—*Dr. Hamilton's Surgical Dispensary at the Medical College*, 1848.

*Enlarged Breast.*—Mrs. ———, aged 38 years, from Steuben Co. Mrs. ——— had her first child when 21 years old, soon after which she had a milk abscess. It was opened near the nipple several times, and left a permanent retraction of the nipple. From that day to this it has uever been perfectly sound, a small induration having remained. Two years ago, (it was then not larger than a pullet's egg) Goodrich, a cancer doctor, applied a caustic; after which it rapidly enlarged, suppurated, and finally cicatrized; but still continuing to increase, it has at length involved the whole breast. The breast is now a large, irregular, and pretty hard mass, slightly attached to the parts underneath, the skin at the upper part somewhat discolored and diseased. It is not much painful, or tender. The glands in the corresponding axilla are enlarged and also some of the absorbent glands just above the clavicle; they are quite hard. Mrs, ——— is *enciente.* Her health is tolerably good. Cancer is not hereditary in her family.

Dr. Hamilton admitted that in this case some difficulty existed in the diagnosis. Some of the signs indicating a "chronic mammary tumor," and others leading to the suspicion that it may be scirrous. It resembles a "chronic mammary tumor" in the early period of life at which it commenced, in its slow growth until interfered with by the quack, in the little pain attending its progress, in the lobulated feel of its surface, in its having attained so great a size without prooceeding to suppuration, in the slight attachments it has formed to the muscles underneath, in the condition of health which the patient presents. While it presents characters of a scirrous in its hardness, the recent implication of the integument at the upper part of the tumor, in its obstinate increase. To these circumstances we should add the enlargement and induration of the absorbents in the neck and axilla. An enlargement simply, might not excite our suspicions, as it may occur in connection with any form of disease of the breast, from mere irritation, but the *induration* is indicative generally of malignancy. The retraction of the nipple, so generally observed in scirrous, is also here present, but from its history we learn that the original abscess was so near the nipple as by its cicatrization to have produced this.

On the whole we believe this was originally a simple sarcomatous tumor, existing in the substance of the mamma, but by meddlesome interference it has began to assume signs of malignancy.

*Treatment.*—If it is really malignant, it has made too much progress to be arrested by an operation. If it is not malignant there cannot be sufficient apology for extirpating it in her present condition. In short, no operation can be recommended at present. Dr. H. recommended a spare diet, and the application of a cicuta plaster, which, he remarked, enjoys with some surgeons considerable reputation for cancerous and scrofulous affections, but its value is overrated.

*Perieal Fistula.*—Charles Clark, aged 12 years, from Conesus. In April last he fell astride a box and struck upon his perineum, which ruptured the urethra, and in a few days resulted in a fistula. It is now about nine months since the accident, and as the fistula does not close, and a firm and close stricture exists in front of the fistula, it was determined to operate.

*Operation.*—A silver catheter was carried down to the stricture, and upon the extremity of this an incision was made, and it was extended backward about an inch and a half until it had passed the whole strictured portion of the urethra. The silver catheter was now withdrawn and a large sized flexible catheter introduced.

Dr. H. remarked that he had always found this a much more difficult operation than lithotomy. The smallness of the contracted channel for which you seek, the fibrous and almost cartilaginous condition of the structures in its vicinity, (in consequence of the ulceration &c.) and the fact that the urethra is generally drawn to the right or left by adhesions, are the chief causes of embarrassment. After the operation is made also, the process of cure is long and tedious. The catheters have to be withdrawn and replaced by fresh ones daily, or at longest every second day. The practice recommended by some surgeons of permitting them to remain undisturbed three or four days, is highly reprehensible. The bladder being now in an irritable condition, phoshatic deposits are made upon the catheter very rapidly, and if it remains in the bladder more than forty-eight hours it will become so encrusted as to lacerate the urethra in its withdrawal, and sometimes it is removed with great difficulty and not without danger of its being broken off within the bladder. The urine also softens and rots the gumelastic catheter, and a new catheter should be used each time.

The case was heard from about the first of March, and it was then, under the care of Dr. Patterson, healing kindly, and the channel of the urethra was free and unobstructed.

*Painful affection of the ring finger and amputation.*—Mrs. Bartlett, of Geneva, aged 40. A small point at the extremity of the ring finger of the left hand, has been exquisitely painful and tender when touched for many years, The spot is not more than two or three lines in diameter, but when touched a severe lancinating pains shoots up the hand. There is an evident enlargement at this spot, the skin being elevated about two lines and very slightly discolored. Mrs. B. is in pretty good health, is not subject to neuralgic pains elsewhere, but she suffers so much from this finger, and especially latterly, that she is determined upon having an operation made.

The disease was diagnosed as a chronic inflammation of some terminal nerve, probably induced by disease of the bone in its vicinity. The patient was assured that nothing short of an operation could afford her relief, and that while a mere incision to the bone, or an excision of the pulp of the finger might possibly be sufficient, yet that neither of these operations could give her a guarantee against the continuance of the disease. Amputation of the last phalanx, could alone be relied upon. Mrs. B. unhesitatingly chose the latter. Dr. H. accordingly performed the amputation at the last phalangeal articulation. On dissection of the finger no disease could be discovered except a small exostosis of about two lines projection, exactly under the seat of the tenderness, and which was beyond doubt the source of the morbid sensibility in the nerves. The dissection and the result of the case confirmed the correctness of the treatment. The wound healed kindly, and up to the 4th of March, two months after the amputation, there had been not the slightest return of pain in the stump

*Varus, and section of tendo Achilles.*—Infant child of William Whiting, of Unionville, Phelps, aged 7 months. This case, it was remarked, would illustrate the fact that even at a very early period of life, when the feet were most pliant, the section of the tendons would not immediately restore the feet to their natural position. No one would think of cutting *all* the contracted tendons in a case of this kind; and even if this were done there are still remaining contracted integument, contracted ligaments, and in the case of older children, malformed and malposed bones. To overcome all these impediments, apparatus, time and patience are requisite; often more time and patience than either the surgeon or friends are willing to bestow: and the result is that where the case is undertaken without a proper knowledge of these difficulties, the cure is often left incomplete. The truth is, that in cutting the tendons we remove only one out of the many obstacles. In this case after the tendo achilles is cut on each foot, a proper apparatus must be applied, and if the parents and the physician in whose care it is to be left have sufficient perseverance a cure may reasonable be expected in from six to twelve months. "This is much longer than some books would lead you to have set, but it is no longer than my experience has taught me we ought always to set."

Dr. H, cut both tendo achilles with a small iris knife, and immediately applied a tin shoe of his own construction. The feet could be now brought entirely straight, but they would immediately return to their former position when the constraint was removed.

The patient was left in the care of Dr. How of Vienna.

*Deafness and puncture of the tympanum.*—James P., aged 43 years; an Englishman, and a basket maker by trade; sits much upon a damp floor and is constantly handling wet willow; he is also much exposed to colds when he goes into swamps to cut osiers. Thirteen years since he had a fever and about a year after he became partially deaf, since which time the deafness has been gradually increasing.

A very critical examination was instituted to ascertain the cause of the deafness, and the conclusion was that he was made partially deaf in *both* ears by a chronic inflammation of the labyrynth, and that an additional degree of deafness was produced in the right ear by a complete obstruction of the eustachian tube.

He was recommended to leave his present occupation, live sparingly, keep his bowels soluble, put a seton in his neck, and put into each ear a drop of the following mixture each day:

℞. Nit. merc. ung.
Ol. oliv. *aa* ℥i.

Before operating upon the tympanum. Dr. H. said he had operated several times in similar cases, but that although the patients had sometimes declared that their hearing was improved, yet that the improvement had never been very striking. In the present case, as there was doubtless a disease of the labyrynth, the perforation of the drum could not possibly restore the hearing perfectly.

The operation was done with a Hey's cataract needle, and four distinct punctures were made, the last of which was enlarged by uotting about one line laterally. The patient complained of no pain in the operation, but only a slight aching afterwards. Each time the needle was introduced the crackling of the tympanum could be heard across the room. The result was about as had been anticipated. Before the operation he could hear the snapping of his finger nails at one foot from the ear; he now could hear the same at two feet.

*Rhinoplasty.*—E. B. aged 14. From an injury received many years since the right ala of the nose is drawn down and the right nostril completely closed,

*Operation.*—A circular piece was cut out of the partition intercepting the right nostril, and to prevent a reclosure a long, narrow linen bag was introduced, and its cavity afterwards crowded with lint. The ala of the nose was then relieved by removing a lunated piece of integument from immediately above and without the ala, and stitching the ala up to the concave edge thus produced.

